# Satisfaction of the quality of life in patients with schizophrenia

**DOI:** 10.1192/j.eurpsy.2021.1429

**Published:** 2021-08-13

**Authors:** R. Ouali, R. Sellami, F. Cherif, S. Elleuch, N. Cheffi, R. Masmoudi, J. Masmoudi

**Affiliations:** Psychiatrie “a” Department, Hedi Chaker Hospital University -Sfax - Tunisia, sfax, Tunisia

**Keywords:** quality, schizophrénia, satisfaction

## Abstract

**Introduction:**

Schizophrenia is a chronic mental disorder that has a significant impact on quality of life satisfaction in patients with schizophrenia.

**Objectives:**

The objective of this study was to examine the impact of socio-demographic factors and psychotic symptoms on quality of life satisfaction in patients with schizophrenia.

**Methods:**

Participants were outpatients of Hedi chaker University Hospital Center in sfax, Tunisia, recruited between January and July of 2019, diagnosed with schizophrenia or schizoaffective disorder. A Demographic questionnaire, the Positive and Negative Syndrome Scale (PANSS) and The Quality of life satisfaction and enjoyment Questionnaire (Q-LES-Q) were administered in this study.

**Results:**

50 patients were included in this study with an average age 40,80 ±9,7. The majority of patients were single (72%), unemployed (60%), without medical heredity (80%) and living with their families (92%). The average score of the positive symptom scale (PANS) was 17.46 (SD = 9.1), the negative symptom scale (PANS) was 12.35 (SD = 7.4) and the psychopathological scale (PANS) was of 27.83 (SD = 14.7). the higher the score of the positive symptom scales (p <10-3) the negative scale score (p <0.002) and the psychopathological scale (p <0.001) was high, more the quality of life satisfaction score has been altered.

**Conclusions:**

Improving the quality of life satisfaction of these patients through these different parameters could be a goal of care complementary to the objectives of traditional care.

